# 1202. Chagas Disease Awareness in a Hispanic Community of the Greater New Orleans Area

**DOI:** 10.1093/ofid/ofab466.1394

**Published:** 2021-12-04

**Authors:** Marcela Hincapie, Kerlly Bernabé, James DeCuir, Julie Thompson, Mariana Avendano, George Nawas, Eric Dumonteil, Claudia Herrera, Margarita Echeverri

**Affiliations:** 1 Xavier University of Louisiana, New Orleans, Louisiana; 2 Tulane University, New Orleans, Louisiana

## Abstract

**Background:**

Chagas disease is a neglected tropical disease responsible for severe disease burden in Latin American countries (≥ 6 million cases). It is increasingly reported in the Southern United States, with an estimated 89,410 human cases (nationwide estimate: 300,000), many of them acquired in endemic zones in Latin America.

**Methods:**

Cross-sectional study to assess the change in knowledge about Chagas disease and triatomine vectors among Hispanic immigrants living in the Greater New Orleans area. All consented participants answered the baseline questionnaire, then received a short video presentation, and completed a post-test to evaluate change in knowledge. Consents, online questionnaires and training were administered in Spanish and English, as needed. Frequencies were computed to describe differences in demographic variables and questions in the pre-posttest. Data was analyzed with R software.

**Results:**

A total of 64 adults (66% women, median age 58 years) completed the pre-post tests and attended the educational intervention. Participants have been living in the US for an average of 23 years and represented 11 countries. Majority were born in Honduras (27%) followed by Nicaragua (16%), United States (13%), Colombia (11%), Ecuador (9%), Guatemala (6%), Mexico (6%) and other counties (12%). Most participants recalled exposure to Chagas disease vectors. Although in the pre-test about half reported ever seeing a Triatomine, less than 20% correctly identified one of three images of a Triatomine provided in the questionnaire. Knowledge about how the disease is transmitted to humans increased from pre to posttest. While higher percentages of men (80%) than women (69%) answered correctly at the pre-test, in the post-test higher percentages of women (97%) than men (95%) responded correctly. In addition, 98% of participants reported that the presentation was clear, 85% would like to learn more about Chagas Disease, and 100% would like to be screened.

**Conclusion:**

Results indicate the positive impact that an educational intervention may have on the knowledge about the disease. Considering the high percentage of Hispanic immigrants in US, increasing awareness of Chagas disease may contribute in the prevention and early detection of the disease among this high-risk population.

**Disclosures:**

**All Authors**: No reported disclosures

